# Dynamic Spatial Tuning Patterns of Shoulder Muscles with Volunteers in a Driving Posture

**DOI:** 10.3389/fbioe.2021.761799

**Published:** 2021-11-24

**Authors:** Jason B. Fice, Emma Larsson, Johan Davidsson

**Affiliations:** Department of Mechanics and Maritime Sciences, Chalmers University of Technology, Göteborg, Sweden

**Keywords:** volunteer data, spatial tuning, shoulder muscles, human body models, volunteer testing

## Abstract

Computational human body models (HBMs) of drivers for pre-crash simulations need active shoulder muscle control, and volunteer data are lacking. The goal of this paper was to build shoulder muscle dynamic spatial tuning patterns, with a secondary focus to present shoulder kinematic evaluation data. 8M and 9F volunteers sat in a driver posture, with their torso restrained, and were exposed to upper arm dynamic perturbations in eight directions perpendicular to the humerus. A dropping 8-kg weight connected to the elbow through pulleys applied the loads; the exact timing and direction were unknown. Activity in 11 shoulder muscles was measured using surface electrodes, and upper arm kinematics were measured with three cameras. We found directionally specific muscle activity and presented dynamic spatial tuning patterns for each muscle separated by sex. The preferred directions, i.e. the vector mean of a spatial tuning pattern, were similar between males and females, with the largest difference of 31° in the pectoralis major muscle. Males and females had similar elbow displacements. The maxima of elbow displacements in the loading plane for males was 189 ± 36 mm during flexion loading, and for females, it was 196 ± 36 mm during adduction loading. The data presented here can be used to design shoulder muscle controllers for HBMs and evaluate the performance of shoulder models.

## Introduction

The computational human body models (HBMs) used to improve automotive safety increasingly include feedback-controlled active muscles (Östh et al., 2015b; Kato et al., 2018; Devane et al., 2019; Larsson et al., 2019; Wochner et al., 2019). The shoulder is an important area for active musculature, as the position a driver relative to safety systems and possibly their injury risk after a pre-crash maneuver may be influenced by their interaction with the steering wheel via shoulder muscle activity. Current HBM shoulder muscle controllers rely on anatomical descriptions of muscle lines of action to derive muscle load sharing patterns (Kato et al., 2018; Devane et al., 2019), but this may not reflect how humans actually use their shoulder muscles. The goal of this paper was to provide volunteer shoulder muscle and kinematic data to be used for developing and validating shoulder muscle controllers in HBMs.

Feedback-controlled active HBMs commonly activate muscles according to a proportional integral derivative (PID) controller when joint angles deviate from their set-point posture (Östh et al., 2015b). The muscles activated depend on the direction of the joint motion and intermuscular load sharing. In current shoulder muscle controllers, intermuscular load sharing is determined by either fixed grouping of flexors/extensors based on anatomical descriptions (Östh et al., 2015a), assumed contributions based on muscle anatomical descriptions (Kato et al., 2018), or contributions based on the measured lines of action of the muscles of the model (Devane et al., 2019). In the shoulder, muscle activity is essential to maintain joint stability, and thus, it is expected that basing intermuscular load sharing on anatomical data alone may lack important features such as antagonist muscle activity. In shoulder modeling work, authors have found improvements of up to 45% in predicted glenohumeral joint reaction forces compared with *in vivo* measurements when EMG-based intermuscular load sharing was utilized over an optimization approach (Nikooyan et al., 2012). In a similar work, optimized shoulder muscle load sharing was shown to underpredict antagonistic muscle activity in the shoulder (Kian et al., 2019).

Feedback controllers for the head and neck in active HBMs have been further developed using intermuscular load sharing derived from volunteer muscle spatial tuning patterns (STPs) (Larsson et al., 2019; Ólafsdóttir et al., 2019). An STP is a polar plot of muscle activity as a function of dynamic loading or isometric contraction direction. In the shoulder, isometric voluntary STPs have been measured with participants in various upper arm postures (Arwert et al., 1997; De Groot et al., 2004; Meskers et al., 2004). The adopted postures do not reflect a driving posture, while it is known that the line of action and potential muscle recruitment is posture dependent, and thus, these measurements may not be applicable to HBMs of drivers (Flanders and Soechting, 1990; Arwert et al., 1997). Furthermore, it has been shown that dynamic and isometric STPs can differ (Ólafsdóttir et al., 2015), and HBMs for automotive safety research model dynamic situations. Currently, there is a deficit of shoulder muscle dynamic spatial tuning data while volunteers maintain a driver posture.

In this paper, we measured the shoulder muscle activity of volunteers while dynamically perturbing their upper-arm at the elbow in eight directions perpendicular to the humerus while volunteers were in a driving posture. The goal of the paper was to build dynamic STPs for the shoulder muscles. In addition, upper arm kinematics were measured during the perturbations. Finally, as injury rates for females have been shown to be 1.47 to 3.1 times higher than males (Bose et al., 2011; Jonsson et al., 2013), the data will be presented separately for each sex. The spatial tuning data will be valuable for the development of active shoulder muscle controllers, and the kinematic data will be useful for the evaluation of the shoulder complex in HBMs. The improvements in shoulder muscle controllers enabled by the data presented in this paper will ultimately be used to design safer vehicles.

## Materials and methods

Eight male and nine female (Table 1) right-hand-dominant persons with no history of shoulder surgery or ongoing self-reported neck, back, or shoulder pain were included in the study. Participating subjects were reimbursed a gift certificate of 250 SEK in value. Subjects provided written informed consent prior to participating in the study, which was approved by the Swedish Ethical Review Authority (application 2019-05794).

**TABLE 1:** Anthropometric measurements of the participants in the present study. The median, maximum, and minimum measurements are shown for both males and females. Sitting height was measured from the seat base to the top of the head parallel to the seatback (17° reclined from vertical). The lateral epicondyle was used for the acromion to elbow measurement.

	Age (years)	Height (cm)	Weight (kg)	Sitting height (cm)	Acromion to acromion (cm)	Right acromion to elbow (cm)
Male
Median	33	174.9	73.9	89.3	39.0	31.3
Max	52	189.2	85.5	95.5	40.5	41.5
Min	28	168.4	61.2	83.3	36.5	28.0
Female
Median	29	165.4	66.0	85.6	35.0	31.0
Max	38	174.5	73.9	87.9	37.5	33.5
Min	24	155.3	51.7	79.2	31.5	26.0

The first step was to take anthropometric measurements of the subjects (Table 1), and then they were instrumented with 13 pairs of surface electrodes, plus a ground electrode on the clavicle (Ambu Neuroline 720, Ambu A/S, Ballerup, Denmark). Before electrode placement, the skin was shaved and lightly abraded with a razor and then cleaned with rubbing alcohol. We recorded muscle activity from the supraspinatus (SUSPINE), infraspinatus (INFSPINE), anterior deltoid (ADELT), middle deltoid (MDELT), posterior deltoid (PDELT), pectoralis major (PEC), latissimus dorsi (LDORSI), teres major (TERMAJ), upper trapezius (UTRAP), mid trapezius (MTRAP), and lower trapezius (LTRAP). The long head of both the biceps brachii and triceps brachii were also recorded, but these data were not further presented due to a lack of proper normalization data. Electrodes were placed with manual palpation according to published sources (Table 2) (Cram, 2010; Ribeiro et al., 2016). Muscle activity was amplified and recorded using a Grael 4K amplifier (Compumedics Limited, Abbotsford, Victoria, Australia) at 2,048 Hz with a 10- 1,000-Hz bandpass filter and an additional notch filter at 50 Hz for powerline noise. EMG amplification took place at the EMG DAQ, which was near (<1 m) the subject.

**TABLE 2:** Approximate EMG surface electrode placements, including the short forms used in the present paper. Manual palpation was used to find the muscle fibers of interest with the following as a guide.

Muscle	Short form	Electrode placement
Supraspinatus	SUSPINE	Just superior to spinous ridge, 2 cm from acromion
Infraspinatus	INFSPINE	2 cm inferior to spinous ridge and aligned with supraspinatus
Anterior deltoid	ADELT	2 cm inferior from acromion in center of the anterior deltoid belly
Middle deltoid	MDELT	2 cm inferior from acromion in center of the middle deltoid belly
Posterior deltoid	PDELT	2 cm inferior from acromion in center of the posterior deltoid belly
Pectoralis major	PEC	Midway between nipple and clavicle
Latissimus dorsi	LDORSI	On the superior aspect, 2 cm from the lateral border
Teres major	TERMAJ	Just above latissimus dorsi
Upper trapezius	UTRAP	3 cm proximal to the insertion on the scapula
Middle trapezius	MTRAP	Midway between the spine of scapula and spinous process of vertebrae
Lower trapezius	LTRAP	At level of lower margin of scapula, 2 cm from vertebrae

**FIGURE 1:**
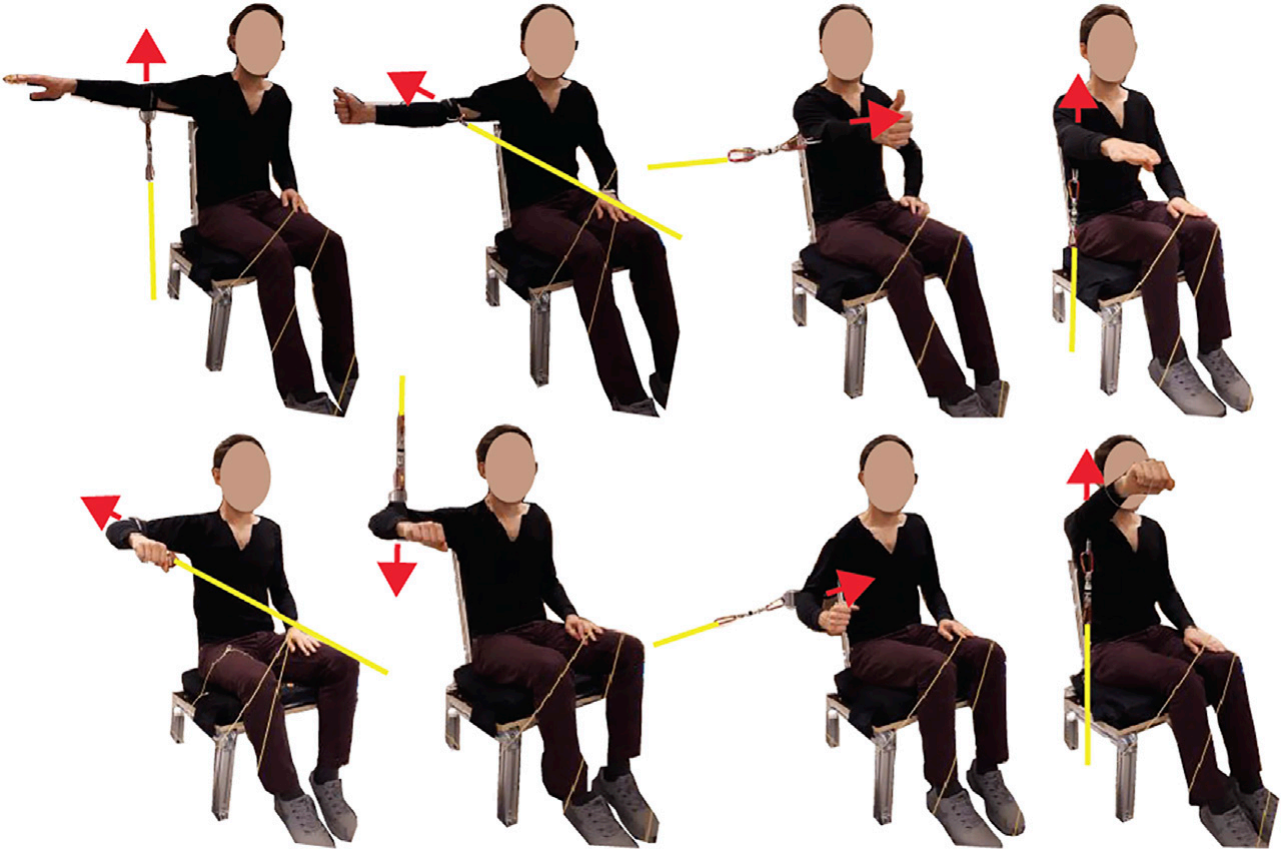
Eight maximum voluntary isometric contraction (MVIC) directions with yellow lines representing a rope that was rigidly fixed at one end and attached to a cuff just above the elbow at the other end, with a loadcell in series. The red arrows indicate a contraction direction. The MVIC directions starting from the top left were arm abducted 90° with palm facing down, maximum abduction; arm abducted 90° thumb pointed up, maximum horizontal extension; arm flexed 90° with the thumb pointed up, maximum horizontal flexion; arm flexed 90° with palm facing down, maximum flexion; arm abduction 90° with the elbow flexed 90° and palm facing down, maximum horizontal extension; arm abducted 90° with the elbow flexed 90° and palm facing down, maximum adduction; arm abducted 30° with the elbow flexed 90°, maximum adduction; arm flexed 125° with palm facing down, maximum flexion.

The first task was to perform shoulder maximum voluntary isometric contractions (MVIC), which were later used to normalize the spatial tuning task EMG recordings. Subjects sat on a custom-built seat with a flat backrest reclined 17° from vertical, and a flat seat pan orientated 90° from the seatback. Their torso was restrained to the seatback with a seatbelt that ran horizontally across their chest under their arms. The maximum contractions were performed by pulling on a fixed rope, which was attached via a cuff just above the elbow, with a load cell in series (TLL-500, Transducer Techniques, Rio Nedo, CA, USA; Figure 1). MVICs were performed in eight directions (Figure 1 for description) with two repeats; the order was randomized within each repetition. Each contraction consisted of a 1- to 2-s ramp followed by 2–5 s of maximal effort. Subjects were given 2 min of rest between MVIC trials. The MVIC directions were chosen based on previous research that showed subjects achieved at least 75% MVIC for each of our muscles of interest, and the average was 89%–95% MVIC depending on the muscle (Dal Maso et al., 2016). In addition to verbal encouragement, as per Dal Maso et al. (2016), subjects also viewed real-time feedback of the loadcell force readings for motivation. The loadcell signal was recorded at 2,048 Hz using LabVIEW and a DAQ system (NI-6356, National Instruments, Austin, TX, USA). For synchronization, the DAQ system generated a 50-Hz pulse that flashed an LED that was recorded by a light sensor on the Grael EMG amplifier.

**FIGURE 2:**
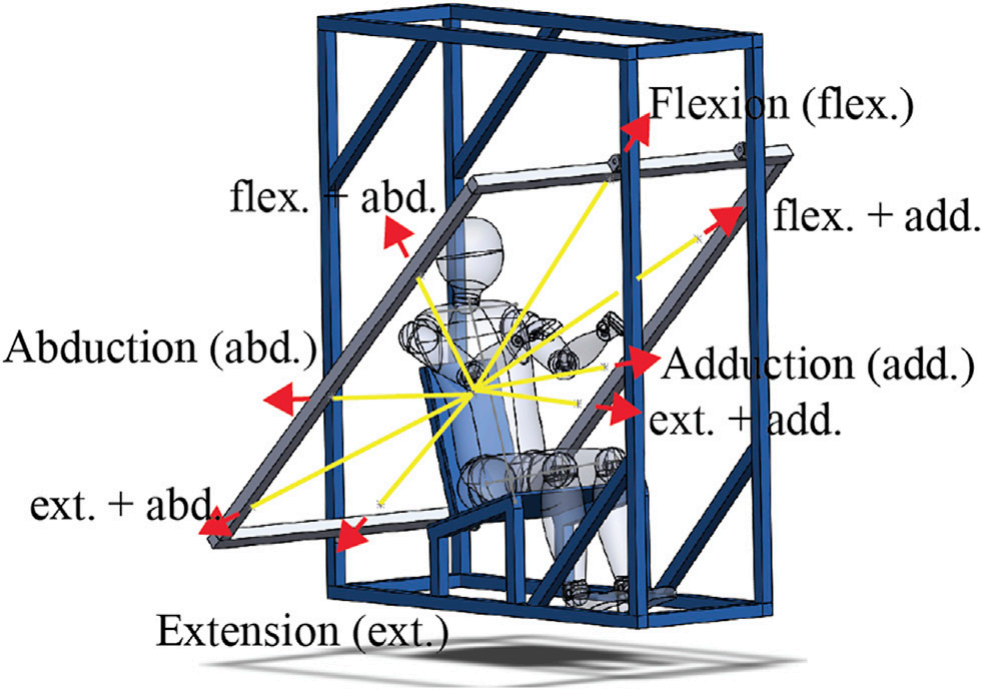
Illustration of the spatial tuning task rig with yellow lines indicating a partial path of the ropes used and the red arrows indicating the loading directions with their abbreviations labeled. The full path of the eight ropes used started at the elbow fixture, ran through a pulley on the frame aligned with the loading plane (shown in gray), which diverted the rope behind the subject, where it went through a second pulley allowing it to be attached to the release mechanism. At the end of each rope, there were two loops. One loop to be attached to the release mechanism such that when the weight was attached, and all eight ropes were held taught with the elbow at a specific location. The second loop on one rope, referred to as the active rope, was attached to the weight. When the release mechanism (a sailing cleat) was opened, seven of the ropes became free, and the active rope was pulled by the now falling 8-kg weight. Note that the weight free fell 2–3 cm before engaging the active rope. Selecting the active rope before release allowed the eight loading directions to be applied without the subjects knowing the loading direction.

The dynamic spatial tuning task was performed next. In this task, eight ropes connected to a polycarbonate elbow fixture worn on the right side of the subject were fed through a series of pulleys on the experimental rig to a release mechanism (sailing cleat), while only one of these ropes, referred to as the active rope, loaded the upper arm (Figure 2). Before loading, the elbow of the subject was held at a specific location determined by all eight ropes being taught when weight was applied to the release mechanism. This arrangement ensured that the arm position prior to each perturbation was consistent. The load was generated when a weight (8 kg) connected to the active rope was released and allowed to drop. By changing the active rope, eight loading directions could be achieved without letting the subject know the loading direction for a trial. Subjects were first fit to the rig by adjusting the amount of stiff foam on the seat to ensure that their elbow was at the specified height, while their upper arm angle was at 43° from vertical with both the upper and lower arm aligned with the sagittal plane. The angle of the upper arm was visually confirmed using a large protractor with a lockable blade. The right elbow of the subject was tightly strapped into the polycarbonate fixture, maintaining their elbow angle at 130°. A load cell (TLL-500, Transducer Techniques, Rio Nedo, CA, United States) was placed between the weight and the active rope. Subjects were also fitted with markers (25-mm-diameter white foam sphere) that were tracked with three visible light RGB cameras (Cameras: UI-3160CP-C-HQ Rev.2.1 Lens: Tamron, 25HB, 12 mm, 2/3”, IDS GmbH, Obersulm, Germany). Markers were fit just medial to the left and right sternoclavicular joints, 2 cm below the sternal notch, the C7 spinous process, the acromion, the lateral epicondyle of the elbow (attached to the elbow fixture), the cubital fossa of the elbow, and Lister’s tubercle of the wrist. Camera images were recorded at 50 Hz, and they were triggered using the DAQ synchronization pulse. The images were captured via USB using LabVIEW (National Instruments, Austin, TX, United States) and IDS APIs (IDS GmbH, Obersulm, Germany).

With instrumentation complete, subjects were exposed to upper arm perturbations in eight directions in a plane perpendicular to their upper arm (Figure 2). In the loading plane, the load directions were 0° (abduction), 45° (flexion-abduction), 90° (flexion), 135° (flexion + adduction), 180° (adduction), 210° (extension + adduction), 270° (extension), and 315° (extension + abduction). Three repetitions of each direction were performed, with the order randomized within each repetition. Subjects were blind to the direction of the applied load and were only aware of a randomly generated 10- to 20-s window for the weight to drop. The preweight drop instructions given to the subjects were as follows: look straight ahead and relax, support the weight of their right arm, do not attempt to anticipate when the perturbation would arrive, and avoid unnecessarily tensing their arm and shoulder muscles. During the trials, the instructions were to pull their arm back to the starting posture as quickly as possible once they felt the force of the active rope and then attempt to hold their arm at the starting posture for 5–10 s. Reminders of these instructions were given at least three times during the task. A rest of 1–2 min was given between perturbations.

The primary goal of this paper was to build shoulder muscle dynamic STPs. The first step in the data analysis toward this goal was bandpass filtering the MVIC and STP EMG data within 30–500 Hz using a dual-pass eighth order Butterworth filter and then taking a 20-ms moving window RMS. In the MVC trials, peak RMS for each subject muscle was defined as the peak value recorded over all MVIC trials. These peak MVIC values then normalized the STP RMS EMG. The STP data were then synchronized in time using the onset time of the loadcell signal, which was found using a finite-difference approach (Siegmund, 2001), confirmed visually and manually adjusted in 13% of the cases. The baseline muscle activity was then found 0.75 to 0.25 s before weight drop, and this was removed from the data. To find the mean baseline activity, first each baseline activity of the subject was averaged over the three repetitions of each direction, and the average and standard deviations of these subject values were calculated. The peak normalized EMG from each trial in a period 0–0.5 s after weight drop (corresponding approximately with the first phase toward max elbow point displacement) were found and then averaged over the three repetitions, and these averaged values were referred to as “peak EMG.” The mean STPs were built in polar coordinates using the subject wise average of the peak EMG values for each loading direction as rho, and the nominal loading directions as theta. The standard deviations of the peak EMG values were used to build mean plus standard deviation STPs. The preferred directions of the mean STP for each muscle were calculated as the vector mean of the STPs and then converted to a unit vector. The preferred direction is a representation of the direction in which the central nervous system is most likely to utilize a given muscle (Fice et al., 2018). Separate STPs were calculated for the female and male subjects. One male subject in the third repetition of flexion + adduction had a partial failure of the release mechanism, and this trial was removed from the STP data.

A secondary goal of the present work was to provide kinematic evaluation data for the shoulder complex of active HBMs. First, the video data recorded during the STP task were corrected for lens distortion using a recording of a 40-mm checkerboard pattern printed on a flat board moved in several orientations, which was repeated for each camera. The TEMA software version 3.5 (Image Systems Motion Analysis, Linköping, Sweden) was used to curve fit a radially symmetric distortion model to the checkboard videos for each camera and calculate correction factors, which were applied to all subsequent videos. Next, five images of a calibration fixture, i.e. a board with nine white spheres (diameter = 25 mm) in a known grid at known heights, were taken from the cameras. These images established a common 3D coordinate system for the markers in the three views taken using TEMA. These images of the calibration fixture showed marker tracking RMS accuracy of 1.36, 0.87, and 1.39 mm for the three cameras. TEMA automatic marker tracking was then used to measure the position of our markers in 3D space. Frame-by-frame manual confirmation of the marker tracking led to corrections in approximately 25% of marker frames. Due to time restraints on marker tracking manual confirmation, only the first repetition of each loading direction was tracked. The previously calculated load cell onset was used to adjust the start time, and frames were tracked from 0.5 s before weight drop to 1 s afterward, approximately corresponding to the time most subjects had returned near the start point, with some oscillation afterward not tracked.

After marker tracking, data processing was started by using the lateral epicondyle, the acromion, and the cubital fossa markers to calculate a rotation matrix that rotated with the upper arm. The initial orientation of this rotation matrix 0.5 to 0.1 s before weight drop was calculated by converting to quaternions, averaging (Markley et al., 2007), and converting back to a rotation matrix. Next, a rotation matrix initially aligned with the ground reference frame, but that rotates with the arm, was calculated by multiplying the inverse of the initial upper arm rotation matrix by the upper arm rotation matrix. A measurement of the approximate elbow joint location relative to the lateral epicondyle marker was taken in the ground reference frame as the average distance between the lateral and medial epicondyle using the video data. A virtual elbow joint marker in the ground frame was then calculated using the upper arm rotation matrix and the initial measurement of the elbow joint center. Next, despite the upper torso constraints used, some movement of the torso was noted, but in order to simplify the use of the kinematic data for HBM validation, it was desired to model a fixed torso, and thus, we calculated the virtual elbow point relative to the C7–T1 joint. To do this calculation, a virtual C7–T1 marker in the ground frame was calculated based on a measurement of the midpoint distance from T1 to the midpoint distance between the markers over the left and right sternoclavicular joints. An upper torso rotation matrix was calculated from the T1, right sternoclavicular joints, and sternum markers, and this was rotated to be initially aligned with the ground as described previously. Again, the virtual C7–T1 marker was calculated from initial measurements and the upper torso rotation matrix. The virtual elbow joint location movement could be calculated relative to the virtual C7-T1 marker in the ground frame, and the final step was to rotate these data, so it was aligned relative to the loading plane, i.e. a horizontal plane rotated 43° about a rightward vector. At each step in these calculations, any missing markers or frames were replaced with the frame previous.

The subject wise average trajectory of the elbow joint location was taken as a vector average of the components that lie within the loading plane for each time period. These data were then converted into polar coordinates, where the peak of the mean elbow joint location was calculated as the largest rho value, which was done for each loading direction. To represent the variability of the elbow joint trajectory, we calculated the mean rho and mean theta of the elbow joint location and plotted a standard deviation ellipse (Shaw et al., 2006) according to Eq. 1. We calculated this variability at the time of peak mean trajectory and 0.8 s after weight drop. We choose to analyze the data at 0.8 s after weight drop as it represented a time when most participants in most perturbation directions had returned their elbow through the starting point, while generally, oscillations around the starting point continued after this time. Finally, we calculated the peak elbow point displacement and time to peak displacement of each of the subject and reported the mean and standard deviation for each loading direction.

All data analyses were performed in MATLAB 2017b (MathWorks, Natick, MA, USA).

## Results

**FIGURE 3:**
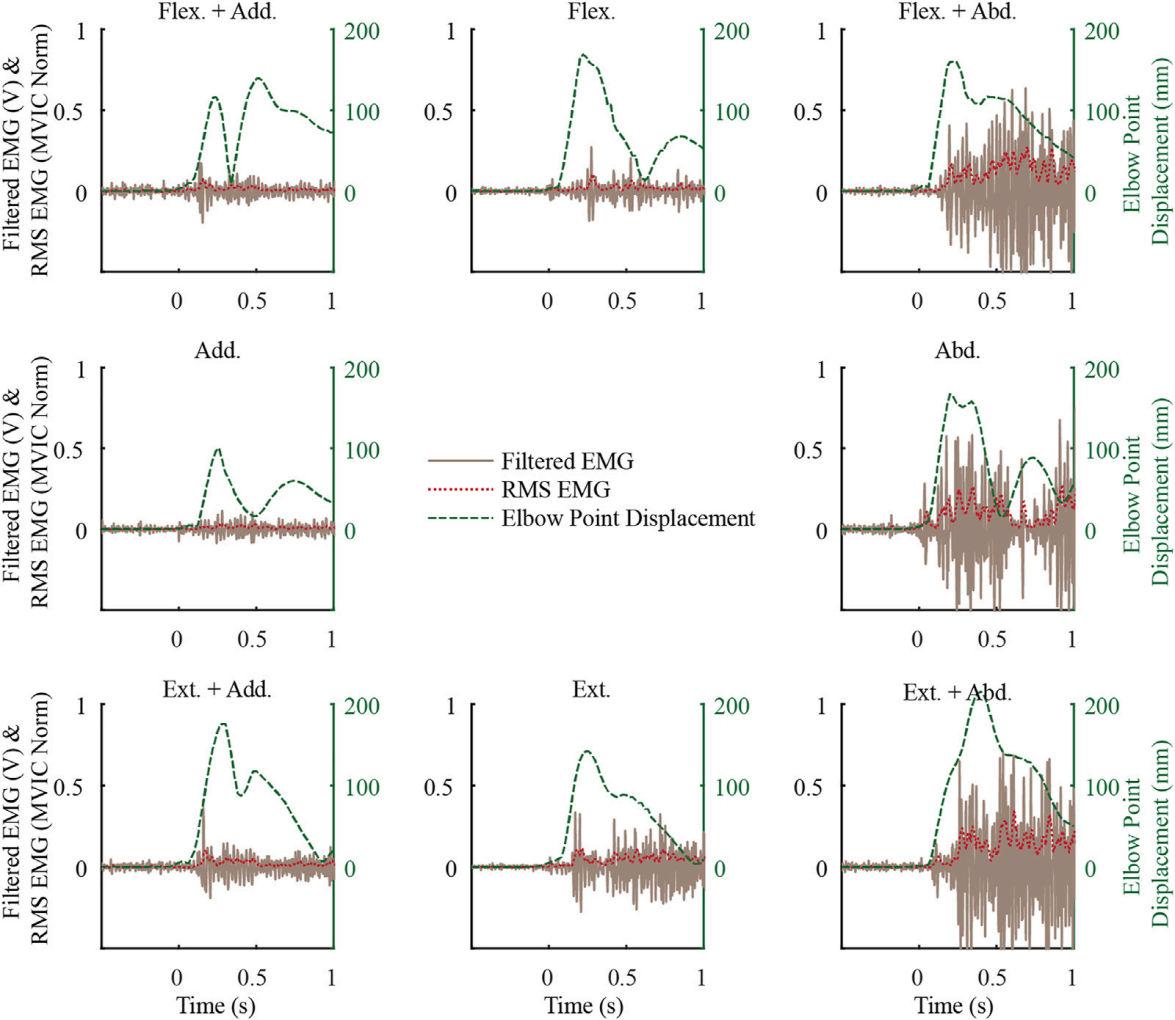
Exemplar middle deltoid (MDELT) muscle activity from a female subject for the first repetition of each loading direction shown along with the elbow point displacement data. See Figure 2 for a description of the direction short forms.

In the exemplar data (Figure 3), muscle activity before the weight drop, i.e. baseline activity, was minimal while supporting the weight of their arm. This trend generally continued, with the mean baseline activity ranging from 0.4 to 3.5 (SD 0.3–2) %MVIC for males and 0.5 to 3.9 (SD 0.3–1.6) %MVIC for females, with the most activity in LTRAP for both males and females. In the exemplar data and more generally, the upper arm began to move almost immediately upon weight drop, and muscle activity followed after a delay. Muscle activity in the MDELT exemplar data varied as a function of loading direction, with the most activity when the load included an abduction component.

**FIGURE 4:**
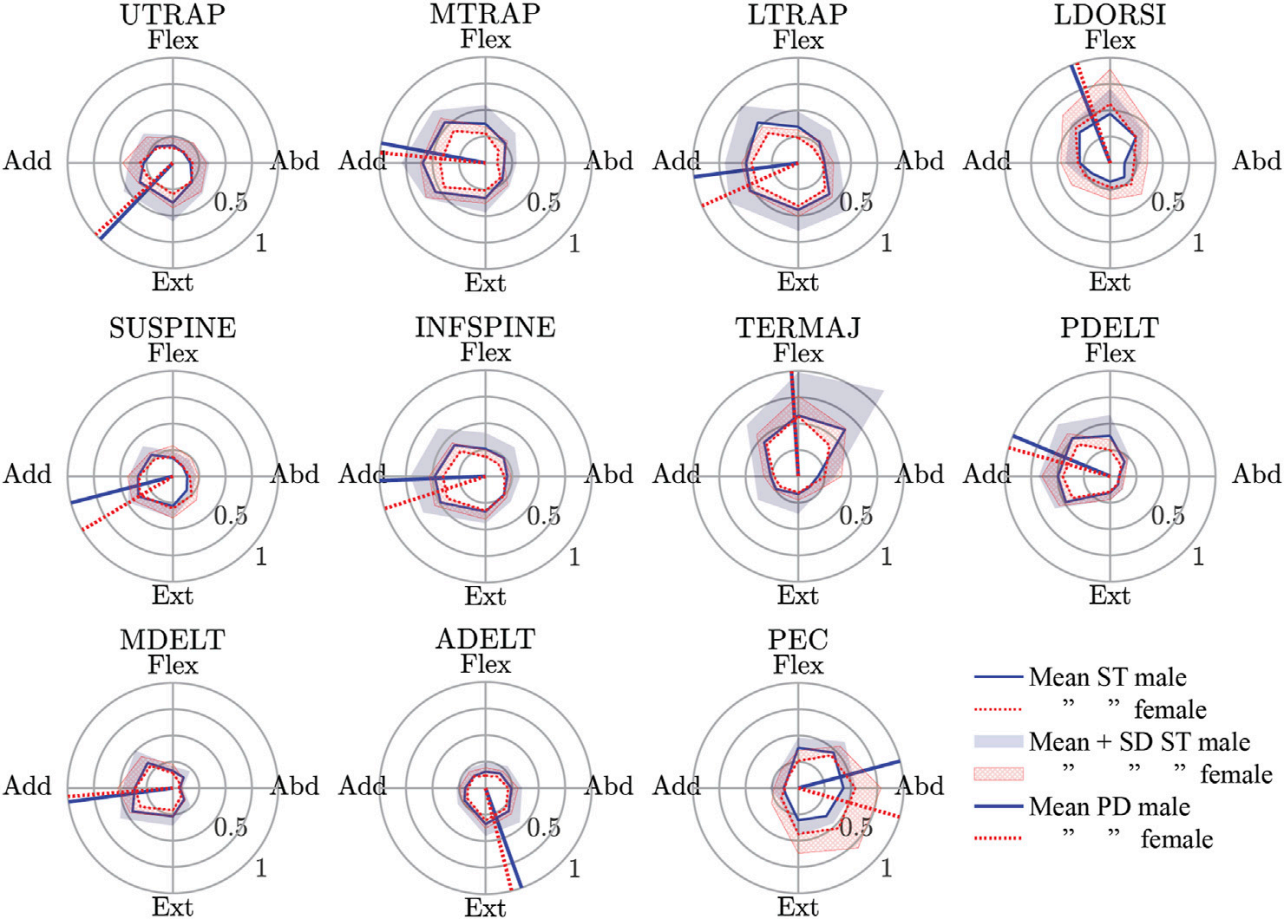
Male and female spatial tuning patterns (STP) for each muscle studied. STP is a polar plot of the normalized muscle activity (rho) as a function of the eight nominal loading directions (theta). The mean STP for both sexes are shown along with the mean + 1 standard deviation of muscle activity in each loading direction. Finally, the preferred activation directions (a vector average of the points in the mean STP) are also shown. See Table 2 for a description of the muscle short forms. Flex., Flexion; Abd., Abduction; Ext., Extension; Add., Adduction.

The spatial tuning patterns recorded for both sexes were visually confirmed to be unimodal, and directionally dependent activity was generally seen for each muscle (Figure 4). All of the muscles studied exhibited some activity opposite of the preferred activation directions, and this is likely evidence for the presence of antagonist muscle activity. The relatively small size of the standard deviation area for the STPs suggests a consistent recruitment strategy of the muscles across subjects, but one possible exception was the TERMAJ muscle. The STPs for males and females were roughly aligned for most muscles. The four largest differences in the preferred activation directions between males and females were 16° in LTRAP, 16° in SUPINE, 15° in INFSPINE, and 31° in PEC.

**FIGURE 5:**
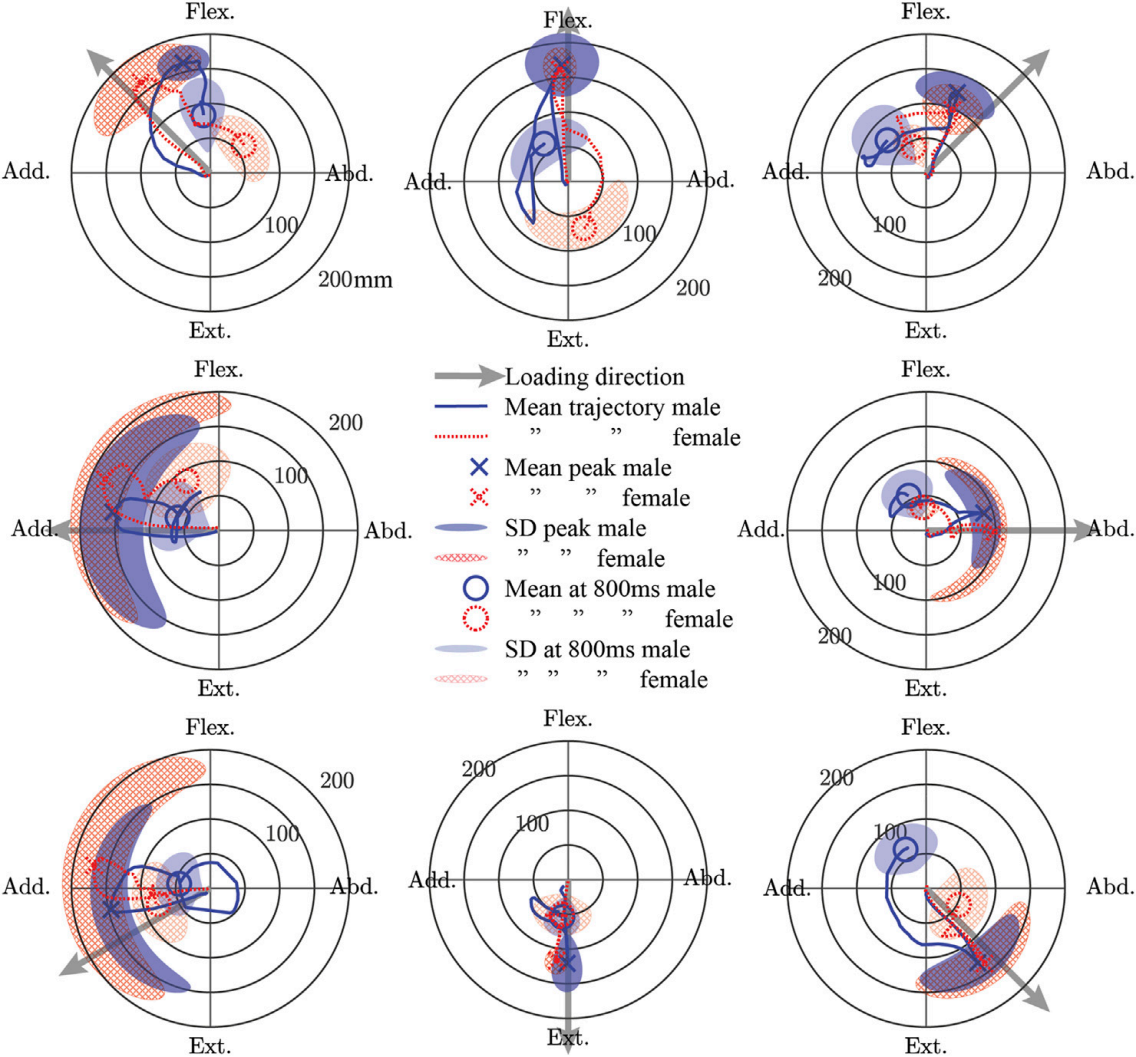
The mean trajectory of a point approximately at the elbow joint center in a plane perpendicular to the initial orientation of the humerus shown separately for male and female subjects in polar coordinates. The peak displacement of the mean trajectory and the displacement of the mean trajectory 0.8 s after weight drop is shown. Furthermore, the standard deviation (SD) of the subject-wise displacements at the time of the peak mean trajectory and at 0.8 s after weight drop are also shown. Flex., Flexion; Abd., Abduction; Ext., Extension; Add., Adduction.

For both males and females, the mean trajectory of the elbow point generally followed the loading vector initially, but the path returning to the start point generally deviated from this vector (Figure 5). For males, the peak of the mean trajectory ranged from 87 (abduction) to 168 mm (flexion). For females, the peak of the mean trajectory ranged from 93 (flexion + abduction) to 176 mm (adduction). The difference in the peak of the mean trajectory between males and females ranged from 22 mm more for females in adduction to 31 mm more for males in flexion + abduction. The most variability in the trajectories at the time of peak mean displacement for both sexes was seen in the adduction and extension + adduction directions. Compared with males, females exhibited more variability in their peak displacements except for the flexion, flexion + abduction, and extension directions. While the magnitudes of displacement at 0.8 s after weight drop were similar for males and females (males: 45–73 mm; females 31–83 mm), the location in polar coordinates at this time varied a lot (difference of 19–166°), except for extension where both sexes aligned (delta 0°).

**FIGURE 6:**
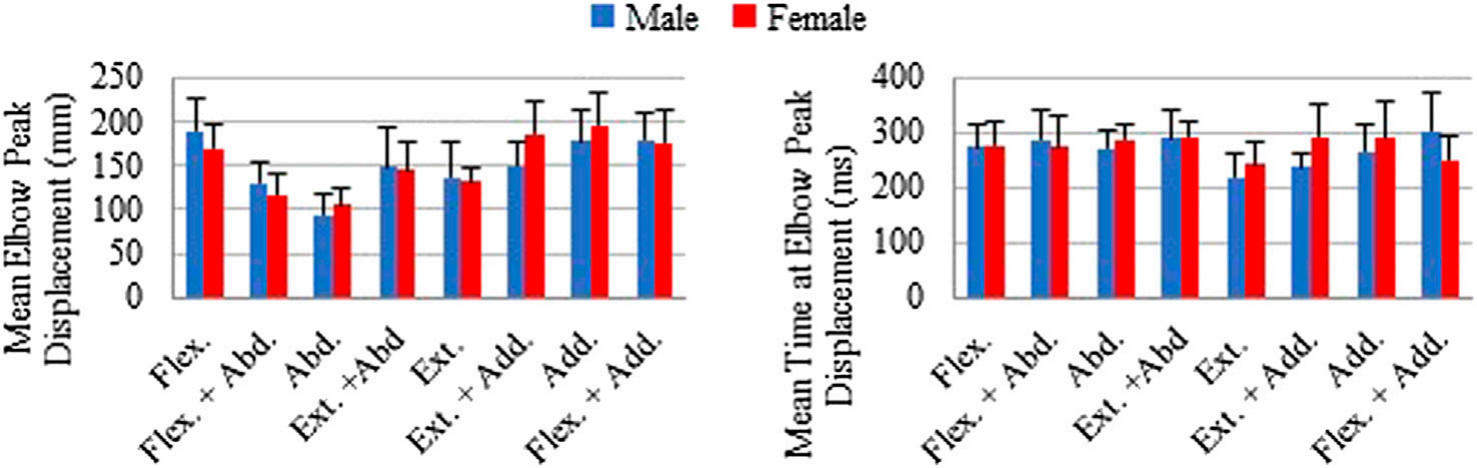
Mean of individual peak elbow point displacements and time to peak displacements shown with plus 1 standard deviation for each loading direction. Male and female averages reported separately.

The mean of individual peak elbow point displacements and the time to reach this peak were generally similar for males and females in all loading directions (Figure 6). The largest elbow point displacements of 196.1 (SD 35.6) mm were in adduction for females and 189.4 (SD 35.9) mm in males. The largest difference in the mean elbow point displacements was 35.6 mm more displacement in females compared with males for the extension + adduction loading direction. The time to reach peak elbow point displacement was similar across directions, with the least amount of time for extension in both males and females. The Supplementary Material contains the complete data to recreate Figures 4–6, together with the SD ellipses at 40-ms intervals, allowing these data to be used to validate the shoulder complex of HBMs.

## Discussion

In this study, we measured shoulder muscle activity in response to dynamic loading applied at the elbow in eight directions perpendicular to the initial humerus orientation, while subjects maintained a driving posture. We found that muscle activity varied with direction for all 11 shoulder muscles studied. The largest difference in the preferred activation direction between male and female volunteers was 31° in the PEC muscle, but generally, the STPs for males and females were similar. We also quantified the displacement of the elbow joint center and found that peak displacements were generally similar between males and females, with the largest difference of 36 mm more displacement for an average female when the arms were loaded in an extension + adduction direction. The data made available in the supplemental material will allow researchers developing HBM to 1) improve the design of their shoulder muscle controllers using the provided STPs and 2) evaluate their shoulder complex using the provided kinematic data.

The preferred activation direction of LDORSI in the present study suggested that it primarily resisted flexion and some adduction loads. However, the anatomical line-of-action for LDORSI implies that it would resist primarily flexion and some abduction loads (Wood et al., 1989a; Wood et al., 1989b; Flanders and Soechting, 1990). Isometric data also showed that LDORSI resists adduction load (Arwert et al., 1997; De Groot et al., 2004; Meskers et al., 2004). An explanation for the discrepancy between the presumed role of LSORSI and its preferred direction lies with its potential role in stabilizing the spine (Gerling and Brown, 2013). Additionally, if muscle activity for a given muscle is driven by other biomechanical constraints (i.e. spine stability) than recruitment outside of the presumed glenohumeral role of the muscle would be possible. More examples of differences in our preferred activation direction and the presumed role of the muscle from anatomy include TERMAJ, resisting primarily flexion loads rather than abduction loads, and ADELT, resisting mainly extension with some abduction rather than extension and adduction. The biomechanical constraints that may explain the TERMAJ result is its role in stabilizing the glenohumeral head (Barra-López et al., 2020). These differences between how the central nervous system used the shoulder muscles and their assumed function based on anatomy highlights the need for physiologically based muscular recruitment in feedback-controlled HBM.

Another approach to simulate feedback control in HBMs is to simulate muscle activation in response to its lengthening beyond a specified setpoint (Östh et al., 2015b; Wochner et al., 2019). This approach attempts to mimic the muscle spindle-mediated stretch reflex in humans, although in humans, the stretch reflex is not always a monosynaptic loop between the spindles of a muscle and its motoneuron pool, which is the most current approach model. The long-latency stretch reflex, in particular, can be a complex context-dependent activation of several muscles including antagonist muscles [see review of Shemmell (2015)]. Basing a muscle controller on STP data will include physiological direction-dependent antagonist muscle activity, as can be seen in all muscles having some activity opposite to their preferred directions (Figure 4). Furthermore, as discussed, some muscles were most active in a direction not aligned with their anatomical line-of-action and possibly not aligned with the direction they were stretched the most. Simple stretch reflex modeling would be the most active when the muscle is stretched the most and would misrepresent this aspect of shoulder muscle control.

Feedback control in active HBMs using STPs has previously been implemented for neck and lumbar muscles (Larsson et al., 2019; Ólafsdóttir et al., 2019). Compared with passive models, these STP-based controllers allowed the response of the model during precrash and crash events to mimic volunteers more closely (Larsson et al., 2019; Ólafsdóttir et al., 2019). In these controllers, the STPs were used to determine the relative contribution of each muscle based on the direction of joint movements. Specifically, the orientation of a neck-to-head or lumbar-based vector projected on the horizontal plane was used to look up the relative muscle activity from each STP of the muscle. A PID controller then determined the overall activity level based on the angular deviation of the lumbar or head/neck vectors from a given set point. Given that the muscle activity data collected in this study showed directional dependency, a similar approach could be adopted in a shoulder muscle controller. The controller would respond to displacements of a humerus-based vector by activating the shoulder muscles according to their STP. This kinematic results of this study could also be used to evaluate a simulation model with active shoulder muscle control.

The preferred directions calculated directly from our dynamic STPs often matched previously published principal directions extracted from cosine function, or second-order polynomial fits to isometric shoulder STPs (Table 3) (Flanders and Soechting, 1990; Arwert et al., 1997; De Groot et al., 2004; Meskers et al., 2004). Some large differences remain, but methodical differences including posture and isometric vs. dynamic loading make direct comparisons difficult. Posture has been shown to be an important factor in the principal directions of shoulder muscles (Flanders and Soechting, 1990; Arwert et al., 1997), but only one study (Flanders and Soechting, 1990) had a posture comparable to the posture used in the present study. Although Flanders & Sochting (1990) used isometric loading similarly to the others, it has been shown in neck muscles that dynamic vs. isometric loading can alter STPs (Ólafsdóttir et al., 2015).

**TABLE 3:** Comparison of literature-reported principal direction vs. the preferred directions reported in the present study. These previous studies report the principal direction from their cosine function or second order polynomial fit to their data rather than the preferred direction calculated directly from the STP used in the present study. Note that the data has been transformed 180° from reported values to match the convention of direction corresponding to the loading direction, rather than the direction of force generated. All data reported in units of (°) with 0° in abduction, 90° flexion, 180° adduction, −90° extension. The present study presents the preferred direction for the males and females separately.

	Flanders and Sochting, 1990	De Groot et al., 2004	Meskers et al., 2004	Arwert et al., 1997	Present study M, F
No. subjects	3: Sex unknown	Unknown	7M 5F	1M 4F	8M 9F
Posture	No. 4 in paper: upper arm 45° from vertical, lower arm horizontal; in sagittal plane	Upper arm horizontal, lower arm vertical; in sagittal plane	Upper arm horizontal, lower arm vertical with horizontal abduction of 30°	No. 2 in paper: upper arm horizontal, lower arm vertical; in sagittal plane	Upper arm 43° from vertical, lower arm 3° flexed from horizontal; in sagittal plane
PEC	2	−25	−18	−23	15, −16
LDORSI	—	156	127	143	112, 108
ADELT	−76	−75	−74	−84	−70, −75
MDELT	−159	−150	−134	−110	−172, −175
PDELT	177	160	−170	162	157, 164
TERMAJ	—	81	107	—	94, 94
INFSPINE	—	−180	−90	−170	−177, −162
SUSPINE	—	—	−90	−141	−165, −149
UTRAP	—	−108	−146	−90	−133, −137
MTRAP	—	168	—	−160	169, 174
LTRAP	—	−155	−145	−155	−172, 156

Male and female data were presented separately because of the higher injury risk for females in automotive crashes (Bose et al., 2011; Jonsson et al., 2013). Historically, an oversized focus on males has been placed in automotive safety research, and thus, validation data for female HBM is very much needed. While the STPs for females and males were generally similar, it remains to be seen if some of the larger differences (i.e. 31° delta in the preferred direction of PEC) would result in important changes in model response. For the kinematic data to be used as validation data, again, there were many similarities for the sexes, but some large differences were noted. Both males and females were most variable at peak displacement when the loading direction was adduction or adduction + extension (Figures 5 and 6). This variability is likely because these directions result in the arm interacting with the body, and body size and/or shape variations may have altered these interactions. The variability in these directions was even larger in females than in males, which may reflect differences in male and female body shapes. In addition, the female elbow point moved 18 mm farther in the adduction loading direction when compared with males. The data presented in the present work is available to model both males and females, but it is unknown if the differences seen are important to model injury risk.

One potential limitation of this study was that the MVIC was not truly maximal. Previous research has suggested that between 4 (Boettcher et al., 2008) and 12 (Dal Maso et al., 2016) contraction directions were necessary to achieve a maximum contraction in each of our muscles. Eight MVIC directions were chosen as a compromise to avoid the risk of fatigue based on pilot data, and the directions chosen were expected to achieve at least 75% MVIC and, on average, 89%–95% MVIC for each muscle (Dal Maso et al., 2016). Another limitation was the lack of proper normalization data for the long head of both the biceps brachii and triceps brachii. Another possible limitation was the use of surface electrodes to record shoulder muscle EMG. The difference between surface and indwelling electrodes at high levels of activity was found to be small or not significant in latissimus dorsi, infraspinatus, or supraspinatus (Waite et al., 2010; Johnson et al., 2011; Allen et al., 2013; Ginn and Halaki, 2015). In contrast, these authors found that surface electrodes generally overestimated latissimus dorsi, infraspinatus, and supraspinatus muscle activity at lower intensities. Data are not available for the other muscles studied. The effect of overestimating muscle activity at lower intensity would see less focused STPs. It is recommended that future work be conducted with indwelling electrodes to understand their effect on dynamic shoulder STPs.

In conclusion, the data presented in this study showed shoulder muscle activity that varied with loading directions, and the spatial tuning patterns developed could be used to determine intermuscular load sharing in feedback-controlled active HBMs. Furthermore, the displacement trajectory of the elbow point during these displacements can be valuable to validate the shoulder complex in HBM. These data are available for males and females, while it remains to be seen if the generally small differences noted justify sex-specific modeling approaches. Ultimately, by providing data to improve the shoulder in HBMs, this work will contribute to the design of safer vehicles.

Equation 1 is a standard equation of an ellipse used to plot the elliptical standard deviation areas in Figure 3 using the data in Supplementary Table S1 of the supplemental material. Phi ranges from 0 to 2π and is used to define the perimeter of the standard deviation ellipse.

$$\{\begin{array}{c} \rho_{ellipse}=\rho_{avg}+\rho_{SD}sin\left(\varphi \right)\\ \theta_{ellipse}=\theta_{avg}+\theta_{SD}cos\left(\varphi \right) \end{array}$$

## Data Availability

The original contributions presented in the study are included in the article/Supplementary Material. Further inquiries can be directed to the corresponding author.
