# Supplementary material for: Dynamic Spatial Tuning Patterns of Shoulder Muscles with Volunteers in a Driving Posture
Source: Front Bioeng Biotechnol. 2021 Nov 24;9:761799. doi: 10.3389/fbioe.2021.761799 (PMC8652075; doi:10.3389/fbioe.2021.761799)
Supplement: Supplementary file 1 [file DataSheet1.PDF]

Supplementary Material

Table S1 – Male spatial tuning data for each muscle studied. EMG activity as a function of loading direction normalized by MVICs. The subject wise mean and standard deviation (SD) that were used in Figure 4 are presented. Normalized EMG presented with 1 equalling MVIC.

	Flex. 90°		Flex. + Abd. 45°		Abd. 0°		Ext. +Abd 315°		Ext. 270°		Ext. + Add. 210°		Add. 180°		Flex. + Add. 135°	
**Muscle**	Mean	SD	Mean	SD	Mean	SD	Mean	SD	Mean	SD	Mean	SD	Mean	SD	Mean	SD
UTRAP	0.168	0.099	0.142	0.132	0.167	0.178	0.256	0.145	0.377	0.179	0.363	0.176	0.277	0.146	0.222	0.173
MTRAP	0.369	0.179	0.263	0.133	0.176	0.116	0.223	0.093	0.330	0.135	0.546	0.114	0.593	0.199	0.543	0.154
LTRAP	0.343	0.145	0.281	0.154	0.249	0.145	0.417	0.227	0.446	0.199	0.528	0.225	0.495	0.202	0.543	0.225
LDORSI	0.468	0.237	0.347	0.099	0.136	0.153	0.190	0.086	0.178	0.094	0.198	0.099	0.289	0.137	0.411	0.115
SUSPINE	0.174	0.064	0.134	0.087	0.136	0.086	0.186	0.063	0.282	0.115	0.380	0.110	0.330	0.088	0.289	0.113
INFSPINE	0.258	0.151	0.243	0.142	0.204	0.123	0.244	0.090	0.331	0.111	0.491	0.188	0.479	0.228	0.414	0.221
TERMAJ	0.578	0.411	0.625	0.526	0.188	0.162	0.146	0.098	0.166	0.188	0.242	0.200	0.262	0.153	0.456	0.234
PDELT	0.384	0.198	0.192	0.068	0.084	0.049	0.107	0.067	0.153	0.072	0.485	0.119	0.496	0.181	0.510	0.189
MDELT	0.162	0.043	0.143	0.085	0.064	0.031	0.151	0.080	0.269	0.087	0.442	0.135	0.362	0.120	0.339	0.166
ADELT	0.155	0.087	0.194	0.099	0.242	0.100	0.307	0.148	0.339	0.112	0.229	0.074	0.191	0.083	0.158	0.094
PEC	0.383	0.095	0.475	0.148	0.429	0.117	0.376	0.094	0.306	0.147	0.133	0.078	0.138	0.131	0.140	0.111

Table S2 – Female spatial tuning data for each muscle studied. EMG activity as a function of loading direction normalized by MVICs. The subject wise mean and standard deviation (SD) that were used in Figure 4 are presented. Normalized EMG presented with 1 equalling MVIC.

	Flex. 90°		Flex. + Abd. 45°		Abd. 0°		Ext. +Abd 315°		Ext. 270°		Ext. + Add. 210°		Add. 180°		Flex. + Add. 135°	
**Muscle**	Mean	SD	Mean	SD	Mean	SD	Mean	SD	Mean	SD	Mean	SD	Mean	SD	Mean	SD
UTRAP	0.143	0.091	0.147	0.126	0.186	0.132	0.247	0.134	0.297	0.131	0.272	0.107	0.314	0.164	0.207	0.142
MTRAP	0.280	0.060	0.169	0.120	0.111	0.065	0.198	0.097	0.260	0.122	0.453	0.195	0.428	0.188	0.425	0.174
LTRAP	0.241	0.071	0.204	0.094	0.234	0.102	0.359	0.099	0.409	0.100	0.443	0.100	0.443	0.096	0.402	0.079
LDORSI	0.560	0.331	0.347	0.166	0.226	0.129	0.286	0.134	0.234	0.113	0.262	0.157	0.306	0.162	0.463	0.174
SUSPINE	0.184	0.106	0.133	0.114	0.160	0.086	0.253	0.064	0.304	0.091	0.359	0.089	0.320	0.109	0.237	0.076
INFSPINE	0.186	0.063	0.167	0.078	0.178	0.064	0.250	0.030	0.323	0.082	0.416	0.137	0.399	0.132	0.334	0.110
TERMAJ	0.569	0.196	0.418	0.221	0.254	0.158	0.138	0.077	0.153	0.072	0.195	0.068	0.231	0.096	0.423	0.136
PDELT	0.251	0.097	0.147	0.084	0.091	0.052	0.098	0.063	0.145	0.076	0.410	0.145	0.450	0.198	0.430	0.143
MDELT	0.143	0.083	0.080	0.053	0.060	0.025	0.111	0.055	0.211	0.060	0.355	0.116	0.356	0.168	0.301	0.124
ADELT	0.116	0.077	0.153	0.074	0.214	0.092	0.271	0.074	0.306	0.075	0.197	0.055	0.167	0.080	0.153	0.064
PEC	0.258	0.100	0.441	0.120	0.542	0.244	0.540	0.265	0.437	0.183	0.185	0.107	0.126	0.104	0.140	0.094

Table S3 – Peak of the mean elbow point trajectory and displacement of the mean trajectory 0.8s after weight drop. Standard deviation (SD) of subject wise trajectories at the time of peak mean displacement and 0.8s after weight drop. Displacements expressed in a plane perpendicular to the initial posture of the humerus (horizontal plane rotated 43° about a rightward vector) using polar coordinates with θ=0° at abduction and 90° at flexion. Use Equation 1 to calculate the standard deviation areas in Figure 5. Flex.-Flexion; Abd. – Abduction; Ext. – Extension; Add. - Adduction

	Flex.		Flex. + Abd.		Abd.		Ext. +Abd		Ext.		Ext. + Add.		Add.		Flex. + Add.	
	ρ(mm)	θ (°)	ρ(mm)	θ (°)	ρ(mm)	θ (°)	ρ(mm)	θ (°)	ρ(mm)	θ (°)	ρ(mm)	θ (°)	ρ(mm)	θ (°)	ρ(mm)	θ (°)
**Male**																
Mean Peak	168.30	93.0	123.97	68.8	86.81	16.9	130.45	-55.4	120.54	-90.8	146.16	-167.9	154.86	170.2	163.09	103.9
SD Peak	45.85	17.8	26.99	28.2	21.92	54.9	28.13	38.7	41.53	10.0	27.36	66.8	46.18	69.9	24.98	13.4
Mean @0.8s	64.56	122.7	72.87	139.9	56.14	116.6	63.52	117.8	54.42	-99.0	45.41	171.6	62.50	162.5	83.58	96.7
SD @0.8s	31.08	57.9	52.48	30.6	36.51	36.0	34.59	42.4	28.81	25.5	33.28	55.4	48.05	41.9	54.10	19.9
**Female**																
Mean Peak	158.81	92.8	93.25	62.7	95.78	-0.3	138.94	-52.3	118.18	-98.3	146.32	-173.8	176.43	160.1	155.18	121.3
SD Peak	27.77	7.7	25.56	26.1	20.04	82.9	30.76	43.0	22.58	10.5	22.73	80.6	36.81	73.8	50.19	30.1
Mean @0.8s	73.75	-71.4	38.44	116.0	30.95	97.7	54.69	-28.7	53.05	-99.0	77.85	-161.2	82.84	123.3	61.26	37.1
SD @0.8s	26.93	70.9	32.06	32.8	15.27	42.4	38.34	55.3	29.56	44.3	36.18	44.0	49.11	44.9	30.54	51.2

Table S4 - Mean of individual peak elbow point displacements and time to peak displacements shown with standard deviation for each loading direction. Male and female data reported separately. This is the data used to create Error! Reference source not found..

	Flex.	Flex. + Abd.	Abd.	Ext. +Abd	Ext.	Ext. + Add.	Add.	Flex. + Add.
**Male**								
Mean Peak Displacement (mm)	189.4	130.1	93.2	149.0	136.8	150.1	178.0	178.9
SD Peak Displacement (mm)	35.9	25.3	24.8	43.8	41.6	26.7	33.8	29.9
Mean Time @ Peak (ms)	275.0	287.5	272.5	290.0	217.5	237.5	265.0	302.5
SD of Time @ Peak (ms)	41.1	51.2	33.7	53.5	45.9	27.1	49.9	68.0
**Female**								
Mean Peak Displacement (mm)	169.5	117.5	105.5	145.6	131.5	185.7	196.1	176.0
SD Peak Displacement (mm)	27.1	24.7	19.1	29.9	14.4	37.0	35.9	36.3
Mean Time @ Peak (ms)	277.8	273.3	286.7	291.1	242.2	291.1	288.9	251.1
SD of Time @ Peak (ms)	42.9	56.6	26.5	30.2	41.8	62.5	67.9	42.6

Table S5 – Male mean elbow point trajectory in a plane perpendicular to the initial humerus posture (horizontal plane rotated 43° about a rightward vector). In polar coordinates with θ=0° at abduction and 90° at flexion. Data from Figure 5, but down sampled to 25Hz from 50Hz for presentation. Flex.-Flexion; Abd. – Abduction; Ext. – Extension; Add. - Adduction

Time	Flex.		Flex. + Abd.		Abd.		Ext. +Abd		Ext.		Ext. + Add.		Add.		Flex. + Add.	
(s)	ρ(mm)	θ (°)	ρ(mm)	θ (°)	ρ(mm)	θ (°)	ρ(mm)	θ (°)	ρ(mm)	θ (°)	ρ(mm)	θ (°)	ρ(mm)	θ (°)	ρ(mm)	θ (°)
0.00	3.05	-154.7	5.07	-24.8	3.49	-50.4	3.80	-97.6	10.65	-123.7	7.17	-126.6	2.87	-177.0	3.62	-143.2
0.04	6.19	-127.6	8.09	-67.9	7.90	-71.0	8.88	-92.0	18.85	-124.8	15.27	-148.1	7.73	-160.1	7.99	-140.7
0.08	17.20	120.7	17.67	57.4	13.04	-31.2	21.86	-72.8	41.28	-97.9	46.62	-157.1	28.05	-165.7	27.34	172.1
0.12	52.23	105.5	45.03	61.7	22.40	-11.4	40.77	-60.6	82.09	-92.1	88.11	-160.5	69.87	-172.8	70.53	162.7
0.16	99.08	102.7	79.18	65.0	40.03	15.0	69.72	-53.0	114.80	-90.3	125.27	-164.4	117.27	-177.9	105.20	144.7
0.20	145.53	98.6	112.15	67.7	67.47	19.2	107.50	-53.4	120.54	-90.8	146.16	-167.9	144.58	178.3	134.99	127.1
0.24	167.16	94.1	123.97	68.8	86.81	16.9	127.60	-55.2	112.74	-90.8	138.81	-176.5	147.33	173.6	155.95	114.8
0.28	166.45	92.9	119.98	72.3	81.01	18.3	127.75	-55.5	101.21	-90.6	126.23	172.5	151.60	167.0	163.09	103.9
0.32	152.90	97.2	104.66	71.1	61.03	26.0	115.54	-56.8	84.46	-91.4	113.61	164.3	143.22	161.9	162.04	96.0
0.36	127.62	104.5	83.05	65.9	48.57	44.1	98.80	-60.6	69.39	-98.7	90.00	161.1	125.36	159.0	155.86	96.0
0.40	104.16	116.4	68.09	69.3	43.66	83.3	87.64	-68.8	63.40	-109.3	61.82	160.8	100.03	155.8	145.04	94.2
0.44	85.18	124.9	63.62	95.3	46.15	124.3	81.93	-77.9	65.94	-116.6	43.27	174.8	80.22	154.4	130.16	92.9
0.48	75.94	-168.3	70.23	153.7	49.83	145.8	81.02	-89.7	67.96	-133.3	45.18	-142.6	65.27	157.3	115.61	94.6
0.52	77.22	-137.1	85.66	165.4	53.27	157.9	79.05	-99.1	65.32	-144.0	46.70	-47.7	62.76	-169.2	105.33	96.0
0.56	78.39	-130.2	94.41	166.8	57.20	155.2	74.63	-106.5	60.46	-152.2	45.50	-36.3	66.90	-165.4	100.81	96.0
0.60	76.32	-130.9	94.21	165.5	60.94	148.3	68.60	-117.1	55.28	-151.9	40.69	-10.6	68.76	-169.7	101.70	96.8
0.64	69.67	-135.2	90.41	166.8	64.49	134.7	61.75	-130.6	50.71	-127.9	38.00	74.2	68.09	179.5	103.16	97.7
0.68	65.03	146.1	87.17	172.4	65.77	126.0	58.43	-167.2	48.21	-107.4	37.15	100.7	64.16	158.1	101.06	97.9
0.72	64.38	130.0	82.95	173.0	63.69	122.5	62.15	134.8	49.38	-113.0	37.69	118.4	61.61	125.7	96.29	97.3
0.76	64.21	122.9	77.59	170.6	60.44	119.4	64.35	115.7	52.06	-112.7	39.25	129.6	62.05	115.9	89.83	96.6
0.80	64.56	122.7	72.87	139.9	56.14	116.6	63.52	117.9	54.42	-99.0	45.41	171.6	62.50	162.5	83.58	96.7
0.84	65.41	121.0	71.49	130.2	52.74	112.3	60.50	135.0	55.68	-99.6	52.08	-179.3	64.39	165.1	78.09	100.6
0.88	64.27	104.1	66.33	117.8	49.79	110.5	56.74	157.4	55.82	-100.5	56.74	-178.3	68.52	169.3	75.88	90.9
0.92	64.61	100.6	62.69	102.9	47.15	109.3	53.32	173.8	54.82	-100.2	57.69	179.5	72.23	171.5	76.17	84.3
0.96	70.86	104.2	69.18	93.7	45.14	105.7	49.79	-177.1	53.42	-99.5	55.95	176.8	74.45	169.5	77.19	80.6
1.00	73.99	103.5	69.69	90.3	44.23	104.8	46.53	175.9	53.09	-115.7	51.75	173.0	73.67	167.3	77.66	80.0

Table S6 – Female mean elbow point trajectory in a plane perpendicular to the initial humerus posture (horizontal plane rotated 43° about a rightward vector). In polar coordinates with 0° at abduction and 90° at flexion. Data from Figure 5, but down sampled to 25Hz from 50Hz for presentation. Flex.-Flexion; Abd. – Abduction; Ext. – Extension; Add. - Adduction

Time (s)	Flex.		Flex. + Abd.		Abd.		Ext. +Abd		Ext.		Ext. + Add.		Add.		Flex. + Add.	
	ρ(mm)	θ (°)	ρ(mm)	θ (°)	ρ(mm)	θ (°)	ρ(mm)	θ (°)	ρ(mm)	θ (°)	ρ(mm)	θ (°)	ρ(mm)	θ (°)	ρ(mm)	θ (°)
0.00	2.09	-101.6	3.49	-69.2	2.39	-29.4	2.84	41.6	4.75	-82.2	3.22	-133.5	2.05	122.3	4.71	-107.6
0.04	4.69	-115.2	6.86	-91.2	6.36	-67.2	6.59	-90.8	9.33	-89.9	8.17	-154.4	5.88	178.0	9.36	-126.9
0.08	12.82	100.0	11.00	-20.2	11.40	-70.1	17.31	-71.5	21.94	-96.6	28.48	-170.7	24.46	176.9	34.91	145.1
0.12	41.30	97.4	27.33	68.0	14.98	-28.2	40.29	-61.0	53.94	-96.7	70.51	-170.2	65.53	178.1	77.53	144.2
0.16	82.89	94.7	56.09	60.7	30.53	-5.3	72.47	-53.5	93.79	-98.6	114.67	-171.2	111.09	175.7	118.52	138.3
0.20	127.93	93.2	80.73	59.3	71.33	5.0	107.29	-50.0	118.18	-98.3	146.32	-173.8	150.90	170.3	152.18	135.2
0.24	153.14	92.2	93.25	62.7	95.78	-0.3	132.82	-51.5	117.56	-97.3	162.19	-178.7	170.32	163.6	161.79	127.9
0.28	159.10	94.0	96.44	66.3	97.65	-3.6	139.78	-52.5	107.44	-98.9	168.08	173.2	178.30	156.3	155.18	121.3
0.32	146.76	97.4	87.53	74.1	79.56	-1.8	125.72	-53.0	96.07	-98.5	168.90	167.4	171.51	148.8	127.29	115.5
0.36	122.08	99.2	83.99	99.3	60.02	-1.2	105.21	-52.2	78.77	-98.5	160.11	165.5	162.29	145.5	117.84	110.6
0.40	95.25	95.6	84.30	108.8	44.15	-8.7	91.24	-52.7	69.92	-102.9	147.35	166.2	147.47	147.6	106.49	110.7
0.44	72.59	90.6	84.90	113.2	39.81	-19.9	81.11	-59.1	63.44	-112.8	135.20	167.6	132.77	148.9	95.00	109.6
0.48	66.02	64.2	87.12	115.3	36.06	-2.3	78.17	-65.5	61.75	-119.4	123.74	170.0	119.57	154.3	81.24	108.2
0.52	62.33	57.1	87.58	117.2	36.08	26.1	75.21	-71.2	60.83	-119.1	111.29	170.9	112.08	154.9	72.32	107.2
0.56	57.78	35.1	86.16	118.6	36.01	77.7	72.06	-66.4	58.09	-113.2	102.32	179.6	110.09	153.0	69.76	106.5
0.60	51.11	4.5	79.15	117.4	36.03	102.7	68.98	-61.2	56.11	-108.5	96.08	-178.3	108.30	149.8	69.25	104.8
0.64	49.31	-34.6	73.43	118.6	35.60	103.5	66.70	-55.6	54.88	-92.3	87.89	-177.7	105.30	143.9	67.79	93.8
0.68	51.88	-53.8	65.26	120.2	34.07	103.4	63.70	-52.3	54.85	-94.0	83.76	-177.6	101.18	137.8	66.14	85.7
0.72	62.13	-58.8	54.44	122.1	32.66	105.3	61.50	-50.3	55.16	-95.5	82.17	179.0	95.59	132.8	64.89	65.8
0.76	70.17	-65.8	45.44	117.5	31.61	104.1	56.89	-40.4	54.17	-97.6	80.42	-161.7	88.70	128.6	63.64	49.5
0.80	73.75	-71.4	38.44	116.0	30.95	97.7	54.69	-28.7	53.05	-99.0	77.85	-161.2	82.84	123.3	61.26	37.1
0.84	73.02	-76.8	37.92	144.9	31.04	90.6	54.13	-23.6	51.49	-100.7	73.73	-162.4	78.28	118.2	59.77	64.5
0.88	69.64	-83.5	33.63	140.1	31.91	97.5	53.05	-20.1	49.96	-100.1	70.23	-167.9	75.38	117.0	57.62	83.9
0.92	68.37	-61.5	31.15	131.9	33.87	109.5	52.15	-5.3	48.16	-94.1	66.95	-171.8	73.70	124.9	55.56	93.8
0.96	66.86	-66.7	30.48	137.8	36.47	116.1	52.43	26.1	46.87	-82.5	63.43	-172.8	74.36	139.5	54.07	84.2
1.00	61.62	-47.0	30.51	140.4	38.25	115.5	52.32	24.8	46.72	-110.7	60.20	-171.7	76.74	147.3	52.70	84.7

Table S7 – Male elbow point trajectory standard deviation ellipses in a plane perpendicular to the initial humerus posture (horizontal plane rotated 43° about a rightward vector). In polar coordinates with θ=0° at abduction and 90° at flexion. Down sampled to 25Hz from 50Hz for presentation. Use Equation 1 to calculate the standard deviation areas as per Figure 5. Flex.- Flexion; Abd. – Abduction; Ext. – Extension; Add. - Adduction

Time	Flex.		Flex. + Abd.		Abd.		Ext. +Abd		Ext.		Ext. + Add.		Add.		Flex. + Add.	
(s)	ρ(mm)	θ (°)	ρ(mm)	θ (°)	ρ(mm)	θ (°)	ρ(mm)	θ (°)	ρ(mm)	θ (°)	ρ(mm)	θ (°)	ρ(mm)	θ (°)	ρ(mm)	θ (°)
0.00	3.21	51.2	3.83	67.6	3.26	58.0	3.80	56.4	7.14	45.8	4.52	67.8	1.56	57.1	3.47	52.5
0.04	5.18	58.5	6.26	68.3	6.15	71.3	7.01	64.9	14.33	62.3	9.45	70.4	4.37	76.8	4.75	67.1
0.08	14.52	48.9	12.96	55.2	8.19	71.7	12.86	68.2	19.82	15.7	12.01	68.2	13.07	71.7	14.94	73.7
0.12	25.62	18.1	18.66	43.2	11.04	71.2	19.77	31.0	20.44	12.1	13.12	66.4	27.06	71.7	33.51	74.6
0.16	39.35	21.6	22.33	35.1	19.74	75.5	29.16	38.6	24.82	12.6	20.67	65.1	44.61	72.3	45.44	52.2
0.20	51.47	17.6	28.69	29.0	20.84	60.0	30.67	41.9	41.53	10.0	27.36	66.8	35.19	73.7	32.52	33.8
0.24	48.70	17.1	26.99	28.2	21.92	54.9	29.21	40.1	54.86	10.8	30.67	73.8	27.13	71.8	17.26	21.5
0.28	48.80	18.5	28.50	29.4	25.05	53.5	33.44	37.6	56.58	10.6	30.98	82.7	46.52	69.6	24.98	13.4
0.32	54.22	16.7	34.14	31.6	37.71	53.0	46.11	38.0	49.13	17.7	26.27	75.8	44.79	71.4	38.85	13.7
0.36	51.44	14.6	38.68	33.5	43.85	46.8	54.53	31.6	34.25	35.0	22.21	71.3	40.18	73.3	40.51	14.2
0.40	48.49	15.2	47.76	47.7	40.07	69.9	62.11	17.8	40.52	45.4	26.18	70.1	36.34	72.8	41.88	15.2
0.44	51.47	24.6	58.65	44.2	34.39	65.0	67.16	17.8	43.82	44.1	29.13	50.3	38.72	67.2	38.41	15.4
0.48	54.27	34.9	67.41	44.8	32.93	62.5	67.79	43.8	48.20	45.3	23.68	47.0	44.92	49.1	38.07	15.5
0.52	50.76	42.3	70.26	46.4	35.96	54.9	66.56	49.7	46.23	46.1	24.92	39.1	39.17	43.8	42.35	18.6
0.56	46.55	46.8	74.16	44.2	40.89	47.7	62.53	42.1	42.65	44.9	27.76	34.5	35.95	45.4	42.62	25.0
0.60	47.23	47.2	78.64	43.8	44.04	45.8	55.39	37.3	39.20	40.8	30.04	35.7	37.84	48.3	42.63	24.8
0.64	51.78	48.7	80.54	46.0	44.51	42.1	45.22	35.5	36.21	32.4	28.67	34.6	41.74	51.5	44.52	23.0
0.68	50.12	50.8	76.21	48.9	43.14	38.7	37.77	38.7	32.69	23.6	26.29	34.7	45.14	54.3	46.34	21.5
0.72	39.99	50.0	68.73	48.9	41.57	37.2	34.23	41.4	30.15	25.6	25.81	34.1	46.45	49.0	49.94	21.0
0.76	31.88	55.6	60.56	47.4	38.91	36.3	32.89	42.1	29.48	24.2	29.60	39.8	47.00	42.1	52.02	20.6
0.80	31.08	57.9	52.48	30.6	36.51	36.0	34.59	42.4	28.81	25.5	33.28	55.4	48.05	41.9	54.10	19.9
0.84	38.34	56.4	43.22	28.1	34.71	37.2	38.90	42.5	28.22	25.0	34.82	60.0	47.85	39.4	55.93	19.2
0.88	46.38	49.3	33.07	28.4	33.68	39.2	41.44	42.3	28.05	25.5	36.00	59.3	45.09	37.7	55.06	45.2
0.92	46.48	42.4	26.18	32.9	33.34	41.8	42.32	42.5	28.58	26.1	35.43	56.0	40.67	38.1	54.18	42.8
0.96	39.89	49.1	17.53	31.9	32.32	44.1	40.43	41.0	30.00	27.7	33.29	54.2	35.53	38.3	54.09	39.6
1.00	39.49	47.4	21.70	29.9	31.63	45.2	38.91	37.0	32.78	48.0	31.24	53.8	31.05	36.9	54.37	38.7

Table S8 – Female elbow point trajectory standard deviation ellipses in a plane perpendicular to the initial humerus posture (horizontal plane rotated 43° about a rightward vector). In polar coordinates with θ=0° at abduction and 90° at flexion. Down sampled to 25Hz from 50Hz for presentation. Use Equation 1 to calculate the standard deviation areas as per Figure 5. Flex.- Flexion; Abd. – Abduction; Ext. – Extension; Add. - Adduction

Time (s)	Flex.		Flex. + Abd.		Abd.		Ext. +Abd		Ext.		Ext. + Add.		Add.		Flex. + Add.	
	ρ(mm)	θ (°)	ρ(mm)	θ (°)	ρ(mm)	θ (°)	ρ(mm)	θ (°)	ρ(mm)	θ (°)	ρ(mm)	θ (°)	ρ(mm)	θ (°)	ρ(mm)	θ (°)
0.00	2.02	52.0	5.83	64.6	2.76	64.0	2.56	58.7	3.68	51.2	2.81	55.0	2.05	50.4	6.66	55.1
0.04	2.57	54.9	7.54	54.1	5.01	55.7	4.11	63.4	5.73	52.9	5.81	69.0	3.70	59.5	10.42	64.0
0.08	8.53	26.5	13.27	51.0	6.70	45.8	9.23	35.8	12.04	21.5	13.00	74.8	9.87	78.6	20.49	56.9
0.12	22.59	8.3	23.68	54.8	7.70	59.4	16.23	38.7	23.88	7.1	21.16	74.8	21.04	82.5	25.63	54.7
0.16	32.10	6.6	25.32	33.6	14.47	65.3	22.96	42.1	29.15	11.8	26.75	75.1	32.54	82.6	27.59	51.0
0.20	33.73	6.7	20.22	25.7	21.96	72.6	28.90	43.4	22.58	10.5	22.73	80.6	37.83	82.8	25.15	44.6
0.24	25.37	7.4	25.56	26.1	20.04	82.9	30.72	43.7	20.13	7.2	26.88	84.2	37.16	75.7	37.64	33.5
0.28	31.67	8.4	35.04	26.2	18.94	81.7	30.85	43.3	29.08	11.3	40.21	79.2	36.45	72.2	50.19	30.1
0.32	39.35	10.3	46.84	29.0	24.18	67.9	32.22	44.2	28.22	14.9	45.37	76.6	35.28	69.8	36.08	29.7
0.36	40.03	9.9	42.00	29.1	18.39	66.2	30.21	45.0	25.54	16.5	46.73	75.9	37.13	62.4	29.35	25.0
0.40	38.14	9.8	33.67	32.4	21.27	66.1	33.06	42.9	28.33	14.7	48.26	77.0	45.70	62.0	26.28	26.7
0.44	41.01	32.6	32.83	41.4	21.33	52.3	37.74	29.2	31.96	27.6	51.24	78.0	56.79	59.6	27.76	27.7
0.48	40.06	51.1	30.90	46.8	17.42	48.8	46.11	29.3	30.74	56.1	54.61	77.8	64.84	61.6	30.78	24.5
0.52	33.46	60.9	29.51	42.0	15.51	45.9	49.89	26.9	31.65	58.9	58.36	67.4	63.32	52.6	33.56	23.5
0.56	31.08	67.5	28.95	38.6	14.18	40.7	49.05	40.3	34.28	50.1	57.03	29.3	55.26	36.6	33.81	36.9
0.60	33.95	70.1	32.76	37.5	13.92	37.3	46.74	45.9	37.08	44.3	55.28	36.6	46.72	34.8	31.79	42.1
0.64	35.25	56.5	33.97	37.9	13.16	36.3	44.01	50.3	37.47	38.6	54.61	42.7	41.10	34.1	30.81	45.8
0.68	34.31	55.3	36.50	38.7	13.24	37.6	41.18	54.0	35.38	44.1	50.21	38.0	39.77	34.5	29.82	45.0
0.72	21.64	73.0	38.74	39.2	13.16	38.5	39.25	57.8	33.43	44.9	44.67	38.7	42.04	36.7	28.46	46.7
0.76	16.79	72.8	36.74	41.2	13.95	39.1	38.83	56.6	30.79	45.1	39.57	39.4	45.59	40.5	29.37	48.5
0.80	26.93	70.9	32.06	32.8	15.27	42.4	38.34	55.3	29.56	44.3	36.18	44.0	49.11	44.9	30.54	51.2
0.84	40.43	69.4	28.12	38.6	16.11	45.7	37.16	55.4	29.37	44.1	34.98	46.5	51.60	48.5	32.27	50.8
0.88	50.39	62.3	26.57	37.4	17.67	49.0	36.53	54.7	29.74	43.9	33.42	45.1	52.82	50.1	33.98	48.3
0.92	52.14	66.2	27.52	39.7	20.25	49.2	36.09	52.2	30.02	42.9	31.71	41.1	53.22	53.5	35.45	46.2
0.96	49.91	70.2	27.38	47.2	22.75	47.3	33.88	57.4	29.93	40.1	31.50	39.5	51.96	54.9	36.55	44.9
1.00	47.93	64.6	26.44	44.6	24.40	46.3	31.38	56.5	28.58	46.7	33.40	43.1	50.12	51.9	36.91	44.0
